# Fabrication of Large-Core Multicore Fiber Bragg Gratings Based on Femtosecond Laser Direct Writing Technology

**DOI:** 10.3390/nano15120891

**Published:** 2025-06-09

**Authors:** Xinda Lu, Rong Zhao, Chenhui Gao, Xinyu Ye, Qiushi Qin, Hao Li, Zhixian Li, Meng Wang, Zilun Chen, Zefeng Wang

**Affiliations:** 1 College of Advanced Interdisciplinary Studies, National University of Defense Technology, Changsha 410073, China; luxinda@nudt.edu.cn (X.L.); chenhuigao@nudt.edu.cn (C.G.); yexinyu17@nudt.edu.cn (X.Y.); qinqiushi@nudt.edu.cn (Q.Q.); lihao18c@nudt.edu.cn (H.L.); lizhixian12@nudt.edu.cn (Z.L.); wangmeng@nudt.edu.cn (M.W.); chenzilun@nudt.edu.cn (Z.C.); 2 Nanhu Laser Laboratory, National University of Defense Technology, Changsha 410073, China

**Keywords:** multicore fiber, fiber Bragg grating, femtosecond laser direct writing, fiber laser

## Abstract

We demonstrate the fabrication of the fiber Bragg grating (FBG) in a self-developed Yb-doped seven-core fiber using two femtosecond laser direct writing methods: a grating array inscription method and a plane-by-plane inscription method. The array fabrication method uses the femtosecond laser to directly write a parallel fiber grating array in the core. The plane-by-plane method is implemented by adding a diaphragm in the optical path to precisely control the length of the refractive index modulation line along the femtosecond laser incident direction. Combined with femtosecond laser scanning, a uniform refractive index modulation plane can be inscribed in the core in a single scanning. Based on these methods, we successfully fabricate high-quality high-reflection FBGs and chirped FBGs in each core of the large-core multicore fiber (MCF) with 14 μm core diameters. Both fabrication methods achieve FBGs with reflectivity above 97% at the central wavelength. We report for the first time the fabrication of high-quality, high-reflectivity FBGs in large-core Yb-doped seven-core fibers using the femtosecond laser plane-by-plane inscription method. This work provides a feasible scheme for fabricating FBGs in MCF.

## 1. Introduction

The fiber Bragg grating (FBG) is a passive device formed by modulating the refractive index (RI) of the fiber core [1]. It has the advantages of compact size, lightweight, corrosion resistance, and electromagnetic interference immunity, and it plays an important role in fiber communication, fiber sensing, and fiber laser systems. Multicore fiber (MCF) refers to the fiber structure containing multiple independent fiber cores in a single cladding. In the field of communication, each core of an MCF can be used as an independent channel, which realizes the space-division multiplexing technology of MCF [2,3,4]. In the field of sensing, MCF can be used for temperature compensation and vector-based measurement through distinct cores, thus improving the measurement dimension and accuracy [5,6,7]. In high-power fiber laser systems, MCF increases the effective area of the fiber core compared with the single-core fiber and has the potential to improve the nonlinear effect threshold and laser output power for high-power fiber lasers [8,9]. With respect to the deepening of research on MCFs, one of the important directions is the application of FBGs in MCFs. At present, the writing methods of MCF gratings mainly include the ultraviolet (UV) laser exposure method [10,11], the femtosecond (fs) laser phase mask method [12], and the fs laser direct writing method [13]. Most of the commercial FBG fabrication methods are UV laser exposure methods, which have the advantage of being suitable for mass production. However, the UV laser exposure method has strict requirements on the photosensitivity of the fiber, limiting the types of fibers suitable for FBG inscription. Additionally, the UV laser needs to strip the coating layer and subsequent recoating processes before and after writing FBG, significantly increasing fabrication complexity [14]. The unique inscription mechanism of an fs laser enables flexible fabrication of FBGs in various fibers without requiring photosensitivity [15,16]. In 2017, Yang, K. et al. used an 800 nm fs laser combined with a phase mask to selectively inscribe FBGs into one core of a twin-core few-mode fiber and applied this configuration to directional bending sensing [17]. When employing more complex MCFs such as seven-core fibers for grating inscription, core positioning would present a technical challenge. Fs laser direct writing technology enables flexible inscription of RI modulation areas with customized periodicities and geometries in the fiber. At the same time, the position of the fs laser focus spot in the fiber can be visually observed with the help of the microscopic objective, ensuring that the fs laser is precisely positioned in the predetermined fiber core during the inscription process, which is especially advantageous when FBGs are fabricated in MCF.

In 2018, a research group from the University of Southampton in the UK used the fs laser point-by-point writing method to inscribe four third-order FBGs into different cores of seven-core MCFs [18]. In 2019, Wolf et al. further optimized the writing technique by inserting a seven-core fiber with helically twisted cores into the polished glass ferrule to inscribe FBGs, which allows for high-performance inscription [19]. However, due to the relatively small overlapping integral value of the fiber grating fabricated by the point-by-point writing method and the core mode field, this method is not suitable for fabricating high-reflectivity gratings in large-core MCFs [20].

In this paper, we employ two fs laser direct writing strategies to fabricate high-reflectivity fiber Bragg gratings (HR-FBGs) in large-core (14 μm) seven-core fibers. The MCF is a self-developed Yb-doped seven-core fiber, designed to be integrated as the gain medium in an MCF oscillator in future applications. The fabricated gratings demonstrate reflectivity exceeding 97% at their central wavelengths. Firstly, we implement the first application of an fs laser direct writing strategy (combining point-by-point and line-by-line writing methods) for parallel grating array fabrication in each core of the large-core MCF [21]. Each grating period contains three 3 μm spaced modulation lines to increase the proportion of the RI modulation area in the core. Then, we use a new method that can directly inscribe FBGs in large-core fibers. This method integrates a diaphragm into the optical path and combines it with fs laser scanning to achieve a uniform RI modulation plane within the core in a single scanning [22]. We demonstrate its first application in grating fabrication within MCFs. This method further improves the performance of the grating. Both fabrication methods achieve FBGs with reflectivity above 97% at the central wavelength.

## 2. FBG Fabrication Based on the Grating Array Inscription Method

**Figure 1:**
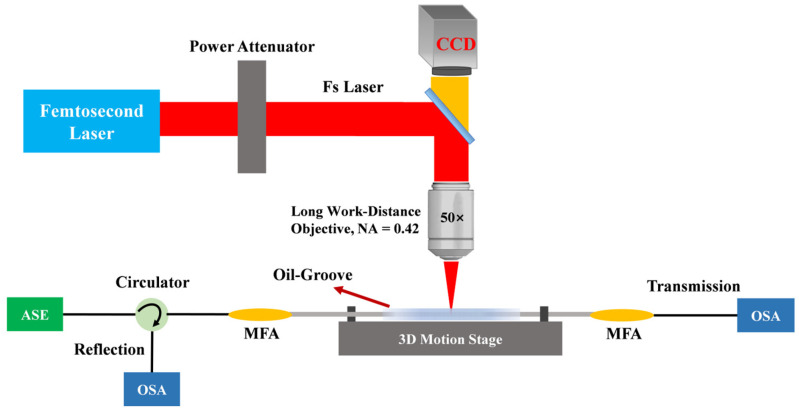
Schematic of the inscription of FBG using fs laser direct writing technology. (CCD: charge-coupled device; NA: numerical aperture; ASE: amplified spontaneous emission light source; MFA: mode field adapter; OSA: optical spectrum analyzer).

The experimental setup for fs laser direct writing is shown in Figure 1. The fs laser is focused into the fiber through a long-working-distance 50× objective lens. The fs laser was produced by BWT Co., Ltd. (Beijing, China) The microscope objective is from Shangguang Instrument Co., Ltd. (Suzhou, China), which has an NA of 0.42 and a working distance of 17 mm. Combined with the movement of the translation stage, it introduces periodic refractive index modulations along the fiber’s axial direction, forming an FBG. The air-bearing stage was customized by Wuxi Geoxin Technology Co., Ltd. Coretech (Wuxi, China). The MCF to be inscribed is placed in a trough containing RI-matching oil to minimize the fiber lensing effect. During FBG inscription, the fs laser is incident into a 50× long-working-distance objective lens and focused into the fiber. The fiber holder is mounted on a two-dimensional computer-controlled pneumatic precision translation stage, which allows the laser focus to be positioned anywhere inside the fiber by moving the stage. The translation stage exhibits a positioning accuracy of ±300 nm and a resolution of 2 nm. The objective lens is mounted on a vertical one-dimensional computer-controlled pneumatic precision translation stage to adjust the *Z*-axis position of the laser focus in the fiber. The numerical aperture (NA) of the focusing objective is 0.42. Additionally, a CCD camera is positioned directly above the objective lens to monitor the grating inscription process and locate the fiber core in real-time. The manufacturer of the camera used in the real-time monitoring system is Nanjing Jingcui Optical Technology Co., Ltd. JCOPTIX (Nanjing, China), and the model is AIC-P231GM-USB.

The optical spectrum measurement system is also shown in Figure 1. The broadband light is injected into Port 1 of the circulator and transmitted to the FBG under test via Port 2. Reflected light from the FBG returns through Port 2 and exits via Port 3 to the OSA for reflection spectrum acquisition. The transmission spectrum can be measured by connecting the output end of the FBG to an OSA via an MFA.

**Figure 2:**
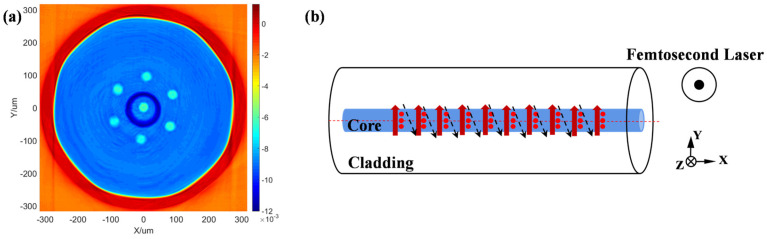
(**a**) The cross−sectional RI profile of the MCF. (**b**) Scanning path schematic diagram of the FBG array fabrication.

A cross-sectional RI profile of the MCF used in the experiment is shown in Figure 2a. The MCF is a self-developed Yb-doped seven-core fiber, with six peripheral cores arranged in a hexagonal ring pattern around the central core. The core diameter is 14 μm, the inner cladding diameter is 250 μm, the core pitch is 45 μm, and the coating diameter is 400 μm. The FBG fabrication method used in this experiment is the grating array processing method. By adjusting the fs laser energy, a uniform filamentary modulation region can be induced by a single fs laser pulse along the incidence direction of the fs laser in the fiber. In the experiment, the single-pulse energy was approximately 2.4 μJ, and the center wavelength of the femtosecond laser was 1030 nm. In each core of the MCF, the grating inscription path is shown in Figure 2b. Here, red and black arrows denote the scanning paths of the focused laser beam. Specifically, the red arrows represent the scanning trajectory when the fs laser is activated, while the black arrows correspond to the path during laser deactivation. By controlling the repetition rate of the fs laser and the movement speed of the computer-controlled displacement stage, the modulation lines form independent lines in the Y-direction of the fiber core. The advantage of the grating array fabrication method is its faster inscription speed compared to the traditional line-by-line writing method. This makes it more suitable for fabricating FBGs in large-core MCFs.

**Figure 3:**
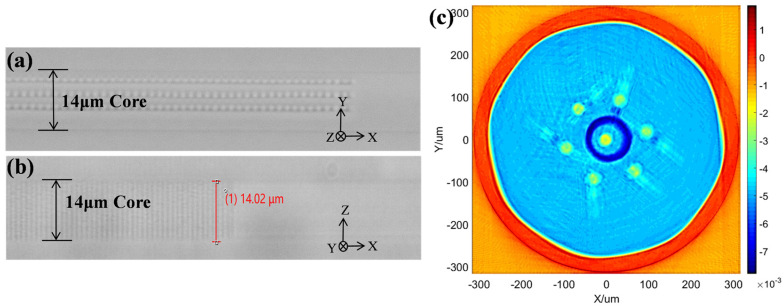
The optical microscopy images of (**a**) the top view and (**b**) the side view of the FBG. (**c**) The RI distribution of the core after the inscription.

In this experiment, the fs laser operates at a repetition rate of 100 Hz with a scanning speed of 0.3 mm/s and a lateral scanning range of 8 μm. This setup allows three modulation lines to be inscribed within each grating period of the fiber core, spaced 3 μm apart. In the X-direction of the fiber core, the grating period of the FBG is set to 1.488 μm, corresponding to fourth-order reflections in the wavelength of 1080 nm. The total length of the grating is 3.4 mm. Figure 3a,b are optical microscopy images of the FBG in the central core of the MCF, captured perpendicular to and parallel to the fs laser incident direction, respectively. Figure 3c shows the RI distribution of the core after the inscription of FBGs. The FBG fabricated using this method exhibits uniform RI modulation in the core along the Z-direction, with the inscription area fully covering the entire core. We provide this figure merely to more intuitively show the RI distribution of the seven-core fiber and to confirm the shape of the grating within the fiber. Due to the defect of the measurement method, the picture cannot be used as the quantitative basis for the refractive index of the fiber.

**Figure 4:**
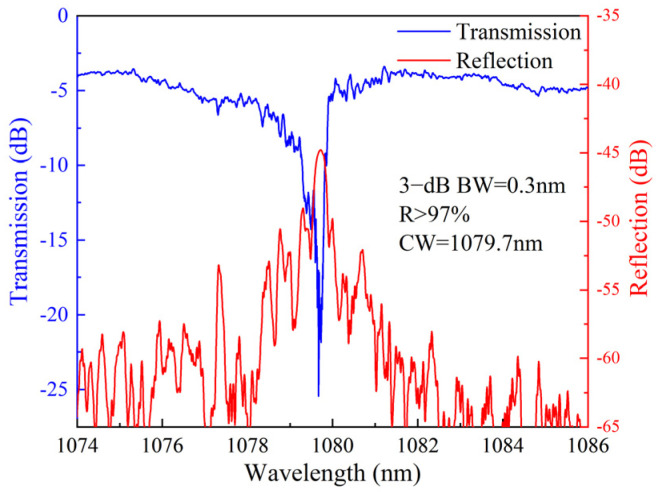
The measured transmission and reflection spectra of the FBG inscribed in the central core of the MCF (BW: 3 dB bandwidth; R: reflectivity; CW: center wavelength).

Spectral measurement results of the FBG inscribed in the central core of the MCF are shown in Figure 4. The reflectivity is greater than 97% at the center wavelength of 1079.7 nm. The 3 dB bandwidth is 0.3 nm.

## 3. FBG Fabrication Based on the Plane-by-Plane Inscription Method

**Figure 5:**
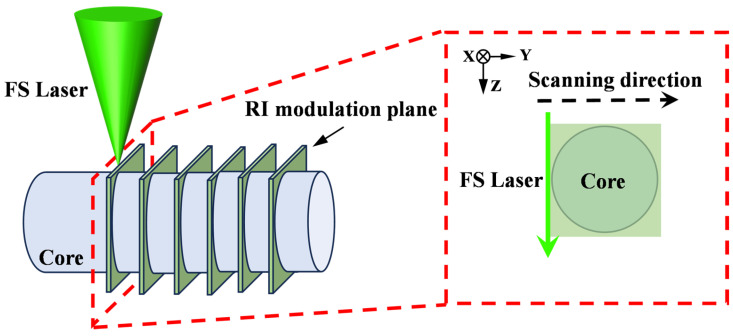
Schematic diagram of fabricating the FBG using the fs laser plane-by-plane inscription method. (Inset: schematic of laser scanning in the Y-Z plane.)

While the grating array writing method enables rapid FBG fabrication in MCFs, its non-uniform RI modulation along the transverse *Y*-axis and asymmetric modulation distribution within cores result in elevated insertion loss. These limitations restrict its capacity to produce long-length chirped fiber Bragg gratings (CFBGs) with both extended total length and enhanced reflectivity. To further improve grating performance, we employed a plane-by-plane fs laser writing method based on beam shaping. By inserting a diaphragm into the optical path to adjust the length of RI modulation lines along the laser incidence direction and combining it with the movement of a high-precision translation stage, this method achieves uniform RI modulation in the transverse direction of the fiber core. The incorporation of an adjustable diaphragm before the focusing objective in the plane-by-plane inscription method is critical for tailoring the spatial characteristics of the fs laser focus. Specifically, reducing the diaphragm aperture increases the Rayleigh length of the laser focus along the propagation direction, which elongates the RI modulation line within the fiber core [20]. A schematic diagram illustrating the fabrication of FBG using the fs laser plane-by-plane inscription method is shown in Figure 5, and the laser scanning path is illustrated in the inset of Figure 5. The green arrow represents the fs laser incidence direction, and the black dashed arrow indicates the scanning direction of the fs laser within a plane.

**Figure 6:**
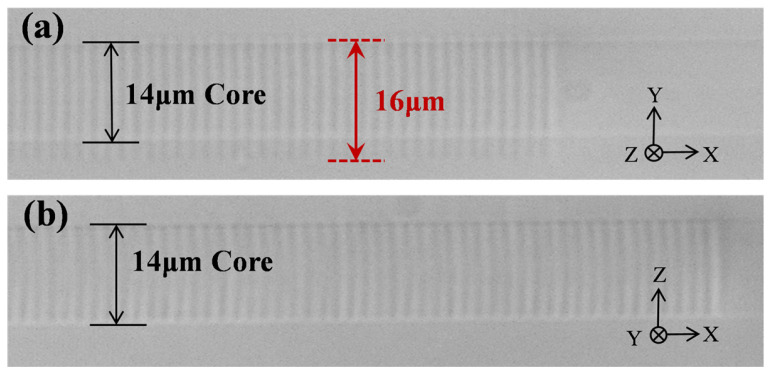
The optical microscopy images of (**a**) the top view and (**b**) the side view of the FBG fabricated by the plane-by-plane inscription method.

In the plane-by-plane FBG inscription experiments, the fs laser operates at a wavelength of 515 nm with a repetition rate of 200 kHz, and the single-pulse energy is approximately 45 nJ. The center wavelength of the femtosecond laser was changed to 515 nm because the 515 nm fs laser has a smaller focal spot, which is better for the precise adjustment of the grating structure in plane-by-plane inscription. A 2 mm diameter diaphragm is incorporated in front of the focusing objective to precisely control the laser beam profile. In this experiment, the fs laser system is essentially identical to that employed in the grating array fabrication method, with differences in the laser wavelength, repetition frequency, and the presence of an aperture before the objective lens. The scanning length is 16 μm, and a scanning velocity of 60 μm/s for each period is used. The grating period is 1.4884 μm, and the total grating length measures approximately 15 mm. Figure 6a,b are optical microscopy images of the FBG in the central core of the MCF, captured perpendicular to and parallel to the fs laser incident direction, respectively. It can be seen that the FBG fabricated using the plane-by-plane inscription method has uniform RI modulation in the transverse direction within the fiber core, and the inscription area covers the entire fiber core. The dimensions of the RI modulation plane are approximately 16 × 14 μm.

**Figure 7:**
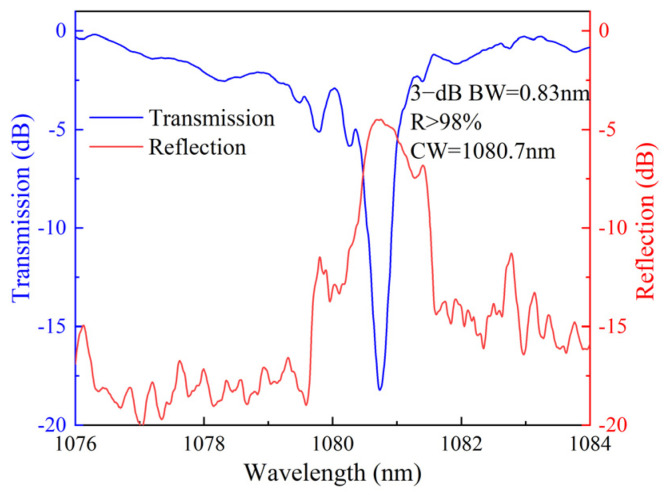
The measured transmission and reflection spectra of the FBG in the central core of the MCF fabricated by the plane-by-plane inscription method.

The transmission and reflection spectra of the FBG are shown in Figure 7. The reflectivity is greater than 98% at the central wavelength of 1080.7 nm, with a 3 dB bandwidth of approximately 0.83 nm. Transmission spectra demonstrate that the FBG fabricated using the plane-by-plane inscription method exhibits lower insertion loss compared to that produced by the grating array inscription method.

To further verify the capability of the plane-by-plane inscription method for fabricating gratings with more periods, we subsequently fabricate a CFBG in each core of the same type of Yb-doped seven-core fiber. The period of the CFBG fabricated in the experiment gradually increases from 1.4854 μm to 1.4872 μm. The chirping rate of the CFBG is 0.88 nm/cm, with a total grating length of approximately 30 mm, corresponding to fourth-order reflections in the wavelength range of 1078.5 nm to 1079.8 nm.

**Figure 8:**
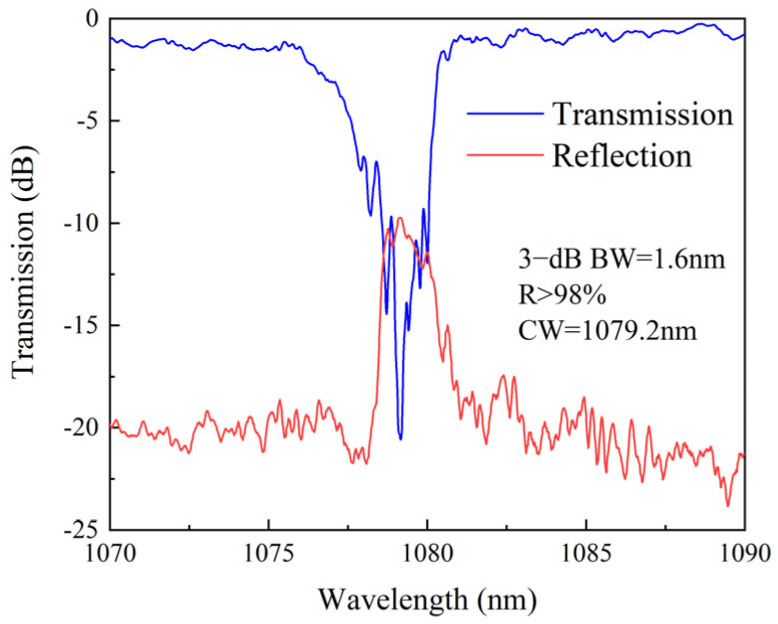
The measured transmission and reflection spectra of the CFBG in the central core of the MCF fabricated by the plane-by-plane inscription method.

The transmission and reflection spectra of CFBG are displayed in Figure 8. The reflectivity is greater than 98% at the central wavelength of 1079.2 nm, with a 3 dB bandwidth of approximately 1.6 nm. The average insertion loss of the CFBG is less than 0.7 dB. This demonstrates that high-quality CFBGs can be fabricated in Yb-doped MCF with 14 μm cores using the plane-by-plane inscription method. These gratings have the potential to serve as high-reflectivity mirrors in MCF oscillators.

## 4. Conclusions

In summary, this work demonstrates two novel strategies for fabricating high-performance gratings in large-core MCFs using fs laser direct writing. Using the grating array inscription method, we fabricate high-quality HR-FBGs in the cores of a Yb-doped seven-core fiber with a 14 μm core diameter. The FBG exhibits a reflectivity exceeding 97% at 1079.7 nm. A key advantage of this inscription method is its ability to enhance grating reflectivity while maintaining a high inscription speed. Then, the plane-by-plane inscription method incorporating diaphragm-based beam shaping was implemented to achieve uniform RI modulation. This approach improved reflectivity to over 98%. Compared to the first method, this approach achieved a significant reduction in insertion loss. Using the plane-by-plane inscription method, we further fabricated CFBGs with a total length of 30 mm and a chirping rate of 0.88 nm/cm, demonstrating broad wavelength coverage across 1078.5–1079.8 nm. The CFBG has a reflectivity greater than 98% near 1079.2 nm and a 3 dB bandwidth of 1.6 nm. This method notably addressed the limitations of asymmetric RI modulation in large-core fibers, improving transverse uniformity compared to the grating array inscription method. We report for the first time the fabrication of high-quality, high-reflectivity FBGs in large-core (14 μm) Yb-doped seven-core fibers using the fs laser plane-by-plane inscription method. These results demonstrate the significant potential of the fabricated multicore fiber Bragg gratings (MC-FBGs) for applications in high-power fiber lasers and multichannel fiber-optic communication systems. Future work will focus on optimizing the inscription process to achieve balanced efficiency and performance in MC-FBG fabrication. Next, we plan to integrate these FBGs into the MCF laser oscillator system. We will evaluate and compare their stability and performance under various experimental procedures, including high-power operating conditions.

## Data Availability

Data underlying the results presented in this paper are not publicly available at this time but may be obtained from the authors upon reasonable request.
